# Patellae as a source of DNA in forensic and archaeological analysis

**DOI:** 10.1007/s00414-024-03363-4

**Published:** 2024-11-08

**Authors:** Živa Miriam Geršak, Aja Golob, Pia Kravanja, Monica Concato, Tamara Leskovar, Irena Zupanič Pajnič

**Affiliations:** 1 Institute of Radiology, University Medical Centre Ljubljana, Zaloška 7, Ljubljana, Slovenia; 2 Institute of Forensic Medicine, Faculty of Medicine, University of Ljubljana, Korytkova 2, Ljubljana, 1000 Slovenia; 3 Department of Medicine, Surgery and Health, University of Trieste, Trieste, 34137 Italy; 4 Centre for Interdisciplinary Research in Archaeology, Department of Archaeology, Faculty of Arts, University of Ljubljana, Ljubljana, Slovenia

**Keywords:** Patellae, WWII, Ancient DNA, Skeletal remains, Genetic identification, STR typing, Forensic, Archaeological

## Abstract

Analysing genetic material from skeletonised human remains has become valuable in forensic and archaeological contexts. While the petrous bone is often preferred for DNA extraction, its availability is not guaranteed, and because of destructive sampling, it is not frequently used in forensic cases. This study explores the potential of patellae as an alternative source of bone material for genetic investigations. Forty-five patellae were sampled from a post-World War II mass grave and an archaeological Christian cemetery dated from the 13th to 19th centuries. A full demineralisation extraction method was used to obtain the DNA, and real-time PCR quantification was used to determine the quantity and quality of DNA. To evaluate the suitability of patellae for forensic and archaeological analyses, short tandem repeat (STR) typing was performed using the ESI17 Fast PCR amplification kit (Promega). To explore the difference in DNA yield, DNA degradation and STR typing success between the post-World War II and archaeological patellae, statistical analysis was performed. The results revealed significantly higher DNA yield and STR typing success in WWII patellae and higher degradation of DNA in archaeological patellae, highlighting the impact of environmental exposure time on genetic material preservation. Almost all WWII patellae achieved a high success rate in STR typing with full profiles generated. More than half of the archaeological patellae showed high STR typing performance and highly informative partial profiles were obtained, indicating the suitability of patellae not only for forensic purposes but also for archaeological genetic analyses.

**Supplementary Information:**

The online version contains supplementary material available at 10.1007/s00414-024-03363-4.

## Introduction

The retrieval and examination of genetic material have evolved into a practical and reasonably attainable approach to extracting insights from human skeletonised remains in the contexts of forensic science and archaeology. In the present day, the analysis of DNA from severely deteriorated skeletal tissues has also become a viable option, enabling researchers to derive distinctive genetic data. Although studies indicate that the petrous bone is the preferred skeletal element type for DNA analysis, its availability for sampling is not always assured [1–12], and because of destructive sampling, it is not often used in forensic cases. It is widely recognized that various types of skeletal elements located in different anatomical regions possess varying quantities of DNA [13–15]. Recent research has revealed that cancellous bones, such as those found in the small bones of the feet and hands (phalanges, tarsals, cuneiforms), and patellae contain higher DNA quantities compared to compact long bones [13, 14] and many of them are anatomically categorised as sesamoid bones, which consist of thin to moderately thick layers of compact bone interspersed with small to large regions of cancellous bone [16]. The body contains numerous sesamoid bones, frequently providing an extra source of strength for muscles and contributing to the stability of tendons. Sesamoid bones, small and often found within muscles or tendons near joints, act as pulleys to reduce stress on those structures. Unlike regular bones that connect via joints, sesamoid bones connect to muscles through tendons [17–20].

The patella is the largest sesamoid bone in the human body, and its typical characteristic is that it is embedded in a tendon. In utero, the patella develops from a continuous band of fibrous connective tissue, which separates into the quadriceps tendon superiorly and the patellar ligament or tendon inferiorly. The patella becomes completely cartilaginous by week 14 and, from then on, forms multiple small foci of ossification. Ossification typically begins at 5–6 years of age when the periosteum quickly forms on the anterior surface of the patella. Its other margins, however, retain a chondro-osseous interface that persists through adolescence [17–25].

Patellae are an example of the sesamoid bones, which have already shown promising results when used as material for analysis in forensic cases [26–29] and archaeology [30–33]. According to a recently done meta-analysis [34] the patellae are not a skeletal element that is often used for genetic analyses. However, the patellae were among the skeletal elements that yielded the most DNA per gram [34]. Patellae have proven to be a reliable source of DNA in a study that tested 55 different skeletal elements in the human body [13]. Patellae were sampled from three complete male skeletons from a 1945 Huda Jama WWII mass grave, sampling 48 types of bones, including the patellae, which showed promising STR typing results. The patellae’s results (2 full profiles, one partial) were comparable to the petrous bones (3 full profiles) and better than femurs (1 full, two partial profiles) [15].

Be that as it may, patellae have not been systematically studied as a potential source of DNA for genetic analyses of aged skeletons in larger quantities, and only a few individual cases have been published; for example, patella was sampled from the mummified body of the Baron Revoltella (1795–1869) and yielded enough DNA for successful identification [35]. The initial study on skeletons from the Huda Jama WWII mass grave had a small sample size for patellae. Therefore, a larger study was conducted to evaluate the patellae’s potential for DNA analysis, including its effectiveness in older archaeological skeletons.

Our study aimed to assess the patella as a valuable skeletal element for forensic and archaeological analysis by comparing the DNA content and quality (DNA degradation) in skeletons of varying ages and burial conditions. In addition, autosomal short tandem repeat (STR) typing success was investigated since STR typing is usually applied for identification purposes to test genetic kinship or to compare reference samples (personal items) with deceased. We compared 20 patellae from a World War II burial site with 25 patellae uncovered at an archaeological site dating from the 13th to the 19th century.

## Materials and methods

### Sample selection and grave description

Samples from a total of 45 patellae were included in the study. They were obtained from two sites, 20 from a post-World War II mass grave and 25 from an archaeological Christian cemetery (the list of samples is shown in Supplementary Material – SM 1 Table 1).

The first site was the Kofin II, a post-World War II mass grave located near Grčarice, Slovenia, which is a mass grave where prisoners of war were executed and dumped in June 1945 following the end of WWII. The skeletal remains were found mixed in a heap of unburied skeletons beneath stones and rocks that had fallen into the shaft. The remains, with the highest density near the entrance, were scattered across the bottom and intermingled in multiple layers in various positions. Most were damaged from the dynamited entrance. Only a small portion allowed individualization, particularly long bones of lower limbs, pelvis, and some spine and ribs. Bones found in pockets or on the stone base were better preserved. Some small hand and foot bones were missing, likely due to water runoff, environmental exposure, or small animals accessing the shaft [36].

The second was an archaeological site in the city centre of Črnomelj, Slovenia. A massive city cemetery was found during renovations of an asphalt parking space area between the church of St. Peter. In an area of 180 square meters, 453 graves were discovered. The graves covered the entire area and were found at a few different layers of depth, from 15 cm to 50 cm. Based on the archaeological finds discovered in the graves (personal items, jewellery, rosaries) and the imperial decree on the burial of deceased outside the city centres in 1806, we can date most of the graves to the 16. – beginning of 18. century. The skeletons were in relatively good condition, placed in the east-west direction, as it is customary for a Christian burial. Most were laid to rest in wooden coffins, as evidenced by the nails and wooden parts of the coffins found adjacent to the skeletons. The stormwater runoff, an antiquated 19th-century sewerage system, and a subsequently added lightning rod collectively contributed to the damage of several skeletons [37].

### Sample preparation

All 45 patellae underwent a thorough cleaning process to eliminate surface contaminants through a mechanical procedure using a rotary sanding tool (Schick, Schemmerhofen, Germany). Subsequently, chemical cleaning was carried out, involving the application of a 5% Alconox detergent (Sigma-Aldrich, St. Louis, MO, USA), followed by rinsing with sterile bi-distilled water and 80% ethanol. The bones were allowed to air-dry overnight. Each bone was divided in half, with the lateral half always taken as the sample for further grinding into bone powder using a sterilized diamond saw (Schick).

To prevent and track possible contamination with modern DNA, special precautions were taken when dealing with aged bones. Cleaning and grinding procedures were performed within a dedicated facility designed solely for processing ancient skeletons inside the microbiological safety hood (Iskra Pio, Šentjernej, Slovenia). All work surfaces, equipment, and the biological safety cabinet were thoroughly disinfected using a combination of bleach, water, and ethanol, followed by an overnight exposure to UV irradiation. This rigorous sanitation procedure was extended to all reagents, laboratory plastics, and equipment for processing the bones, which were all sterilized and UV irradiated. Each sample was processed using clean equipment. Each extraction batch included extraction-negative controls (ENC), and every PCR reaction included negative controls to verify the purity of the reagents and plastics used. Additionally, an elimination database was established. The research project received approval from the Medical Ethics Committee of the Republic of Slovenia (0120–233/2020/3 and 0120–345/2023/6).

### DNA extraction, purification and quantification

The patella parts were individually transformed into fine bone powder utilizing a Bead Beater MillMix 20 homogenizer (Tehtnica, Domel, Železniki, Slovenia). Grinding was conducted for 1 min at a frequency of 30 Hz in metal vials that were cooled with liquid nitrogen before grinding to prevent overheating. Bone fragments were also cooled before powdering. DNA was extracted from half a gram of powder using a full demineralization protocol as specified by Zupanič Pajnič [38]. DNA was purified and eluted in 50 µl of TE buffer using the EZ1 Advanced XL device (Qiagen, Hilden, Germany) and EZ1 & EZ2 DNA Investigator Kit (Qiagen), according to the manufacturer’s instructions.

To assess the DNA content and degradation, a real-time PCR (qPCR) method employing the PowerQuant System (Promega, Madison, WI, USA) was utilized. This method allowed for the simultaneous quantification of total autosomal DNA and male DNA while estimating the presence of PCR inhibitors. To gauge the level of DNA degradation, two targets of varying lengths (short Auto target 85 bp long and long Deg target 294 bp long) are amplified. The Auto/Deg ratio was computed with the PowerQuant Analysis Tool (Promega) and utilized to gauge the degradation of DNA. All samples were quantified in duplicate according to the manufacturer’s recommendations. Amplification reactions were conducted in the Quant Studio 5 Real-Time PCR system (Applied Biosystems—AB, Foster City, CA, USA). The threshold values for the IPC shift and the Auto/Deg ratio were set at 0.30 and 2, respectively, in accordance with the manufacturer’s guidelines [39]. The quantity of DNA expressed in ng DNA/g of bone was calculated for each bone sample.

### Autosomal STR typing

For kinship analysis in forensic and archaeological investigations and for identification through comparison with personal items of deceased in forensic investigations, successful autosomal STR genetic typing is essential [40, 41]. All patellae were typed for autosomal STR loci, and the genetic profiles were used to confirm DNA authenticity by comparison with elimination database profiles. Since the femurs from all Konfin II grave victims were already typed [42] STR profiles from the WWII patellae were compared to check for matching endogenous bone DNA (authentic bone DNA and not contamination of modern DNA). To confirm DNA from archaeological patellae, three successfully typed patellae (samples 250, 256, and 258 - see SM 1 Table 1) were selected. Additional skeletal elements (petrous bones and canines) from these three skeletons were processed to ensure DNA authenticity by matching STR profiles across different skeletal elements.

The PowerPlex ESI 17 Fast System (Promega) PCR reaction with 30 amplification cycles and a 25 µl volume was performed in the Nexus MasterCycler (Eppendorf) according to the manufacturer’s guidelines [43]. PowerQuant Auto target quantification results determined the final PCR input volume of bone extracts. If DNA concentration was 0.058 ng/µl or more, 1 ng of DNA was used for PCR. For concentrations below 0.058 ng/µl, the maximum input volume (17.5 µl) of extract was used. The same volume was applied to ENC samples. For WWII (Konfin II mass grave) and archaeological (Črnomelj archaeological site) patellae the threshold for all dyes was 150 RFU. All elimination database samples underwent autosomal STR typing. STR profiles were obtained using the SeqStudio Genetic Analyzer for HID (Thermo Fisher Scientific), WEN Internal Lane Standard 500 (Promega), SeqStudio Data Collection Software v 1.2.1 (TFS), and GeneMapper ID-X Software v 1.6 (TFS).

### Statistical analysis

The hypothesis that there are statistically significant differences in the patellae from archaeological site Črnomelj and a post-WWII mass grave in the amount of the DNA - expressed in ng DNA/g of bone, degradation ratio - Auto/Deg ratio of the DNA, and STR typing success was formulated.

Normality and homogeneity of variance were tested using the Kolmogorov-Smirnov test with Lilliefors’ significance correction. The research hypothesis was tested using 95% confidence intervals for means or medians, an appropriate method for group differences in medical studies [44–46], with IBM SPSS Statistics for Windows, version 25.0 (SPSS Inc., Chicago, IL, USA). Given the small sample size, confidence intervals may have limited power [46], so hypotheses were also tested using p-values, with significance set at *p* ≤ 0.05.

The database included data from the bones of 45 individuals, 25 from Črnomelj and 20 from WWII mass graves.

One sample from Črnomelj and one from a WWII mass grave lacked data for the quantitative variables, so they were omitted. Four cases (three from Črnomelj and one from the WWII mass grave) had DNA amounts but no Auto/Deg ratio due to no Deg targets being amplified. For these, the Auto/Deg ratio was calculated as Max + SD, resulting in a value of 146.58 (122.65 + 23.93). These calculated values were saved in the database.

The Kolmogorov-Smirnov test showed that data is not normally distributed. Thus, non-parametric Mann-Whitney test were performed, and medians were used for the confidence intervals. The differences were considered statistically significant if 95% confidence intervals did not overlap.

## Results and discussion

### DNA quantification

The results of the PowerQuant average measurement of the duplicated qPCR analysis of WWII and archaeological patellae samples are summarized in SM 1 Table 1. Y, Deg, and Auto targets are expressed in ng DNA/µl of extract, and the amount of DNA (obtained from 1 g of bone powder) is expressed in ng of DNA per g of bone. Data acquired from PowerQuant Auto target amplification were used to calculate DNA yield. No DNA was detected in one WWII patella and one archaeological patella. More than 0.5 pg of human DNA per µl of extract, the minimum recommended for reliable results with the PowerQuant qPCR kit [47], was found in all bone samples except three (see SM 1 Table 1, labelled in red). The lowest amount of DNA (Auto target) was 0.0069 ng/µL, resulting in a partial profile (13/17 STR markers detected). A full profile (17/17 markers detected) was obtained from 0.0147 ng/µL.

In a previous study [48] we found that DNA quality (degradation of DNA) is more crucial than quantity for obtaining full or useful partial profiles. When both Auto and Deg targets were detected, we successfully obtained full or useful partial profiles. An IPC shift value of more than 0.3 indicates that inhibitors are present. In our case, this occurred in two samples - one from WWII and one from Črnomelj (samples labelled in green in SM 1 Table 1). However, no inhibition of PCR occurred, and STR typing was successful in both samples. The degradation of DNA was higher in ancient patellae samples (with values up to 122) than in WWII samples (with values up to 8). For one WWII and three archaeological samples, the degradation index was impossible to ascertain because the Deg target was not amplified. ENC samples did not produce amplification products on any of PowerQuant targets, except the Auto target in ENC 2.

The average DNA yield in WWII patellae samples was 13.18 ng DNA/g of bone, and in archaeological samples, it was 2.82 ng DNA/g of bone. DNA yield was 4.7 times higher in the WWII patellae than in the archaeological samples. Comparing the average degradation index between WWII and archaeological samples, it was lower in WWII than in archaeological samples (5.00 versus 40.92), where degradation was 8.2 times higher.

### STR typing

All WWII and archaeological patellae were genetically typed, 18 out of 20 WWII patellae samples generated full autosomal STR profiles (90% of the samples), and 13 out of 25 archaeological patellae samples (52% of the samples) produced useful informative STR profiles, mostly with more than 12 STRs amplified (see SM 1 Table 1 – ESI17 STRs). Eleven archaeological patellae samples generated profiles with 12 or more STR loci, and two of them produced profiles with 9 and 10 STR loci, respectively. Profiles with at least 11 STR loci amplified can be submitted to the International Commission on Missing Persons (ICMP) database [49]. In sample 3 WWII, we obtained a full profile without drop-outs. For sample 265 Č, there was a drop-out of one STR marker (D2S441), which is longer than 350 bp, indicating DNA degradation. Since the IPC Shift slightly exceeded the value of 0.3 (0.52 and 0.53 – see SM1, Table 1, marked in green), no impact on STR typing was observed. For half of the archaeological patellae samples, extracts with low DNA quantity were obtained, and consequently, for two samples, STR genetic typing failed.

A comparison of STR typing success between WWII femurs and WWI patellae yielded the following results. Of the 62 femur samples, 55 produced full profiles. Among the 20 WWII patella samples, 18 generated full STR profiles. The STR typing success rate of obtaining full profiles was 89% for femurs and 90% for patellae, indicating that their success rates are comparable.

To confirm endogenous bone DNA, genetic profiles of WWII patellae were compared to the consensus profiles of the Konfin II mass grave victims and for all of them, a match to the corresponding victim was found. Endogenous bone DNA in three archaeological patellae samples was confirmed through a comparison of genetic profiles obtained from petrous bones (for all three patellae samples - for samples 250, 256 and 258) and teeth (for two patellae samples – for samples 250 and 258) of the same three skeletons (skeletons from grave 1037, 1092 and 1109). All the genetic profiles obtained from different skeletal element types of the same skeleton matched each other (see SM 1 Table 2). Confirmation of endogenous bone DNA, along with ENC samples showing no amplification products and no match with the elimination database, indicates no contamination of endogenous bone DNA with contemporary DNA. Additionally, the authenticity of DNA obtained from bones was confirmed with high degradation indexes, which are, according to our expectations, higher in archaeological than in WWII patellae samples.

### Statistical analysis

Based on the results of the Mann-Whitney test and confidence intervals, the research hypotheses that there are statistically significant differences in the patellae from archaeological site Črnomelj and a post-WWII mass grave in the amount of the DNA - expressed in ng DNA/g of bone, degradation ratio - Auto/Deg ratio of the DNA, and STR typing success were confirmed.

**Fig. 1:**
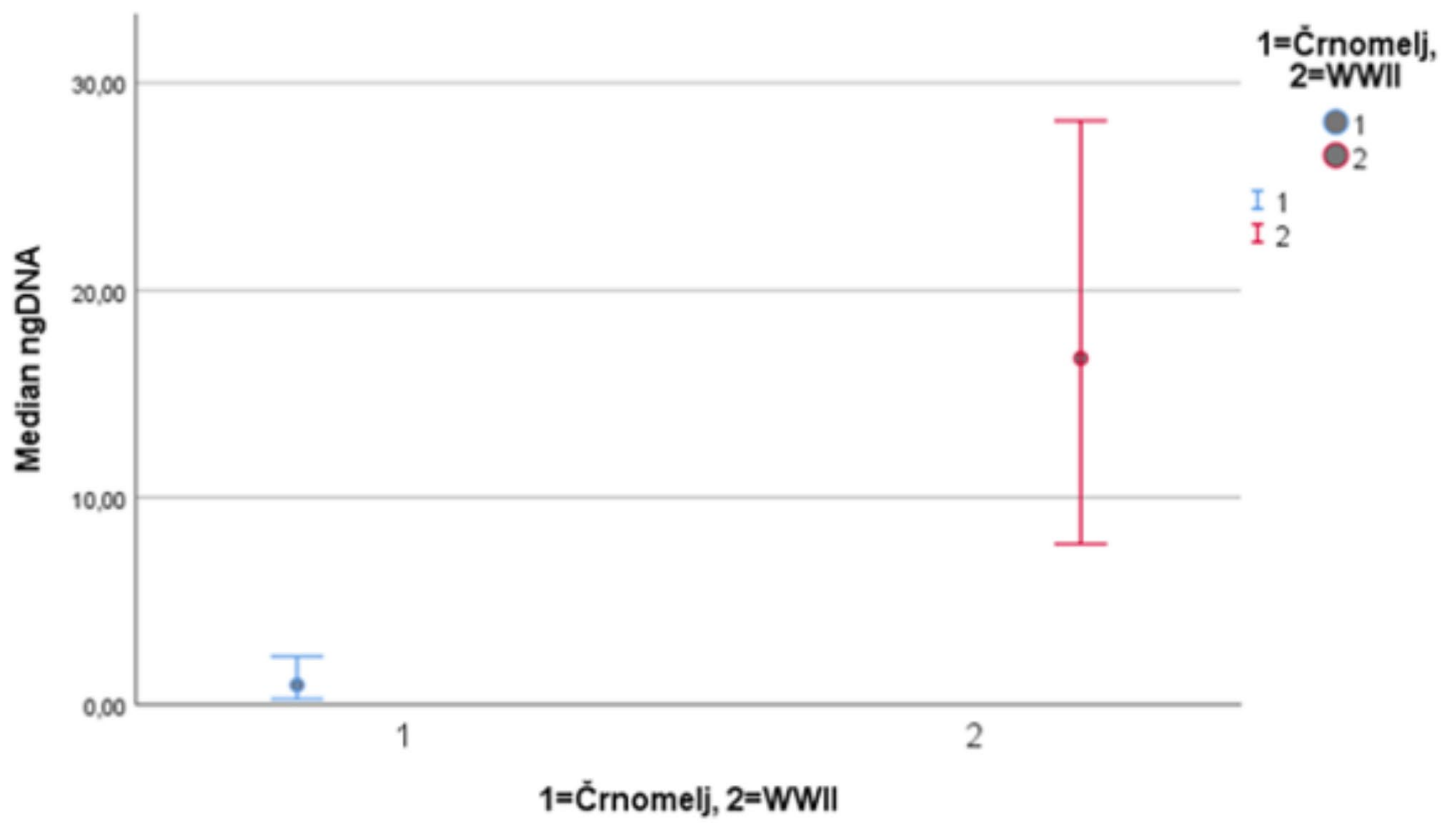
95% confidence intervals for medians for the amount of the DNA extracted from patellae (for archaeological site Črnomelj and WWII mass grave, separately)

**Fig. 2:**
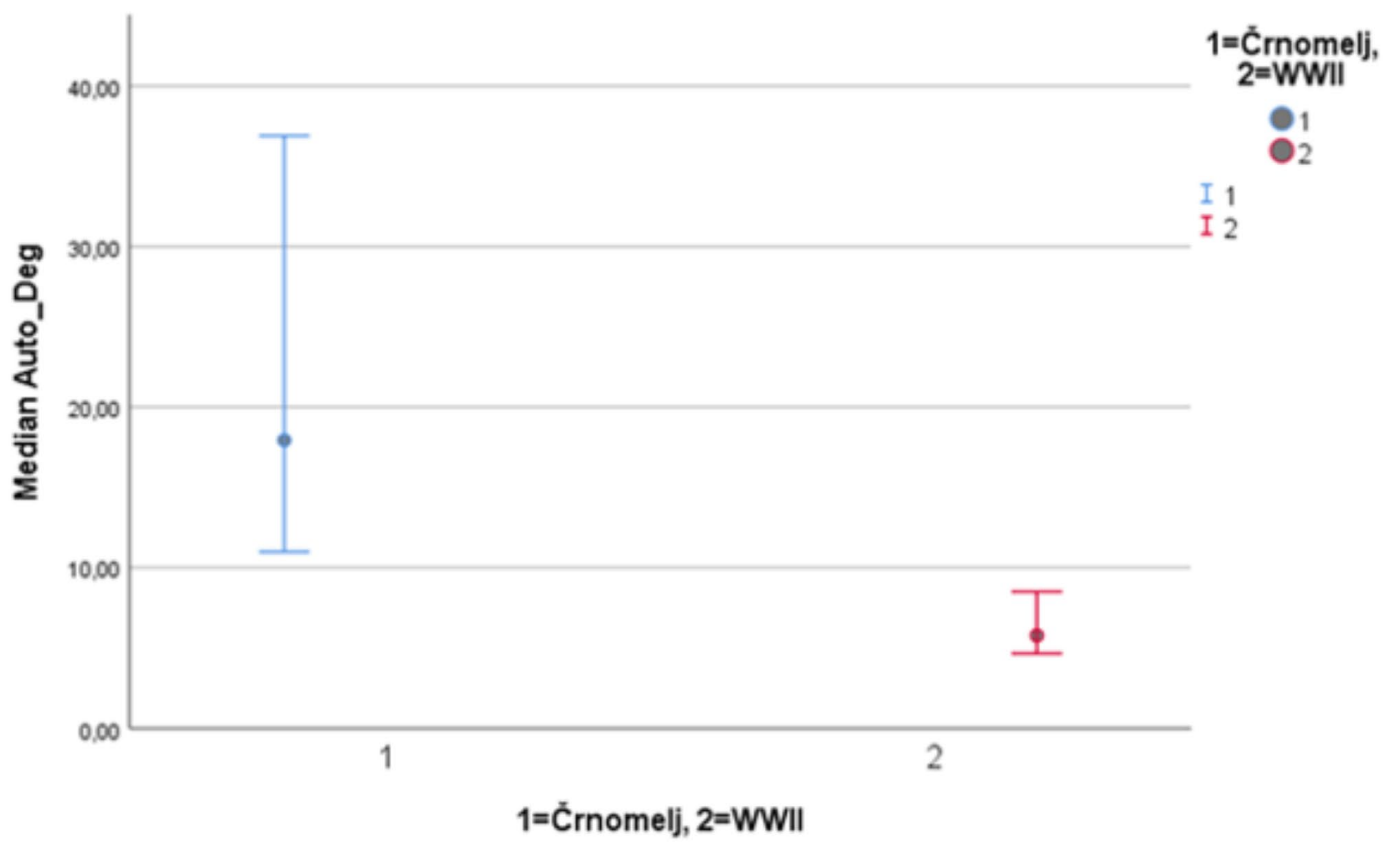
95% confidence intervals for medians for the degradation ratio of patellae (for archaeological site Črnomelj and WWII mass grave, separately)

**Fig. 3:**
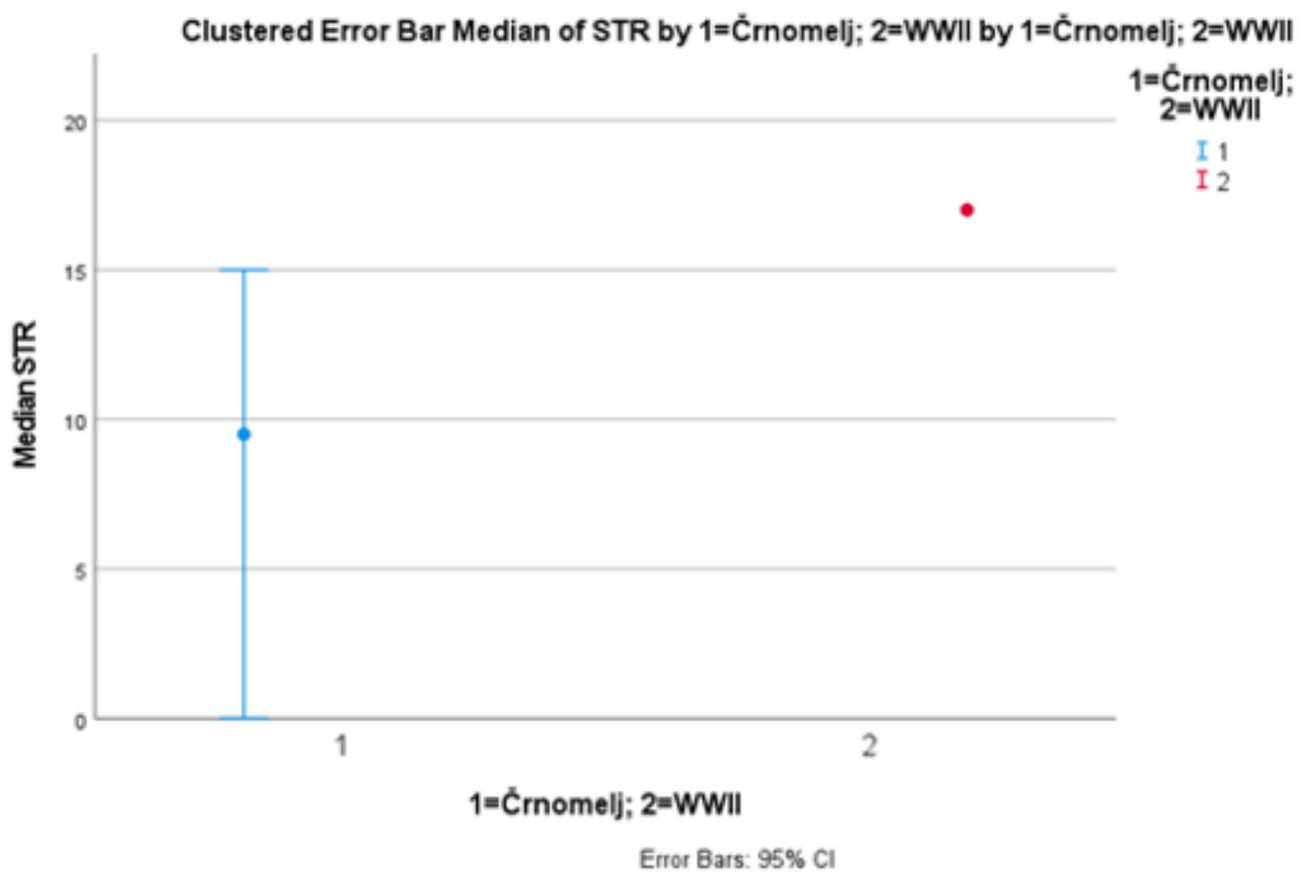
95% confidence intervals for medians for the STR typing success expressed in a number of successfully amplified STR loci in ESI 17 Fast System - Promega (for archaeological site Črnomelj and WWII mass grave, separately)

Results suggest significant differences (*p* < 0.001) in the amount of DNA extracted, significant differences (*p* < 0.001) in the degradation ratio, and significant differences (*p* < 0.001) in the autosomal STR typing success among patellae from archaeological site Črnomelj and WWII mass grave (Figs. 1, 2 and 3). The amount of DNA extracted was higher in patellae from WWII mass graves, the degradation ratio was higher in patellae from archaeological site Črnomelj, and the autosomal STR typing success was higher in WWII patellae (see SM 2).

Our research findings present intriguing insights into patellae’s utility as a source of DNA for genetic analysis in forensics and archaeology.

It is hypothesized that the presence of soft tissue remnants within the trabecular structure of skeletal elements rich in cancellous bone tissue might be responsible for the elevated nuclear DNA yields observed [50, 51]. However, we must be aware that trabecular bone is more prone to decay than compact bone [52], especially in ancient skeletons [53–55].

Our research results show statistically significant differences in the quantity, STR typing success and degradation of DNA extracted from patellae between the two contrasting burial contexts: the post-World War II mass grave and the archaeological Christian cemetery of Črnomelj, confirming our research hypothesis. Notably, the patellae recovered from the post-World War II mass grave exhibited higher DNA quantities and higher STR typing success compared to those retrieved from the archaeological Christian cemetery of Črnomelj, and the quality of DNA was higher (degradation index was lower) in WWII samples. The difference in DNA content, STR typing success, and DNA degradation between WWII and ancient patellae could be attributed mainly to the differences in the age of the skeletons and the time of exposure to decay. There are various other factors that affect DNA preservation, such as, the preservation conditions, soil composition, burial depth, temperature, and other environmental factors as well as the differences in the method of their storage after excavation and the time from excavation to the DNA analysis [52, 56–62]. The preservation of DNA in bone tissue is a question that has been posed many times, and we have not yet arrived at a definitive answer [47, 48, 50, 51, 63–75]. However, ancient DNA is well known to be more fragmented than its younger, modern counterpart and is usually fragmented into between 100 and 500 base pairs [56, 76]. To perform kinship analysis, which is often used in forensic analysis to identify skeletal remains [40, 41, 77–80] and ancient investigations [35, 81–87], successful autosomal STR genetic typing is necessary. Our results showed high STR typing performance in WWII patellae (with full profiles obtained from 90% of the samples) and archaeological patellae (half of the samples roduced informative genetic profiles).

The study was done using half a gram of bone powder for DNA extraction. A previous specific study was done for evaluating DNA extraction from small quantities of bone powder from aged bone samples which results showed that the full-demineralization protocol using 500 mg of bone worked for both WWII and archaeological samples, while the partial-demineralization protocol with a smaller amount of 75 mg of bone powder was only effective for WWII bones [88]. It was concluded that the improved extraction method, which uses less bone powder, speeds up the process, and handles more samples, is suitable for identifying well-preserved aged bone samples in routine forensic analyses. We also reviewed other studies which to improve the efficiency and effectiveness of DNA extraction from bone samples by developing and comparing methods that optimize yield, quality, and practicality, using smaller or bigger amounts of starting material [89–92]. These studies show that while more bone powder can result in a higher total DNA yield, it does not always guarantee better quality of DNA profiles. Larger amounts of bone can lead to higher levels of inhibitors and less efficient extraction if not processed correctly. The newer full demineralization protocols demonstrate that high-quality DNA can be obtained from smaller amounts of bone, with improved efficiency, reduced reagent use, and better overall performance in DNA extraction and profiling. This approach simplifies the process and enhances the reliability of obtaining usable DNA for forensic and historical analyses while also preserving the bone from which it was sampled, which has practical implications in archaeological and valuable skeletons from our history.

The patellae have proven to be effective skeletal samples for genetic analysis in both forensic and archaeological studies. Our research highlights significant potential in utilizing patellae for forensic genetic identification. This is attributed not only to well-preserved DNA but also to simple anatomical recognition, which enables the reassociation of skeletal remains in case of commingling. This becomes particularly valuable in mass graves where fragments of the same skeleton may be scattered, and the remains are not found in their original anatomical positions. Among sesamoid bones, the patellae stand out as easily recognizable due to their larger size and consistent preservation.

## Conclusion

This study contributes valuable insights into the potential use of the patellae as a consistent and reliable source of DNA for forensic and archaeological investigations. The study compared patellae from two burial contexts - a post-World War II mass grave and an archaeological Christian cemetery, revealing significant differences in DNA quantity, STR typing success and DNA quality (degradation index). Patellae from the post-World War II mass grave had higher DNA content, likely due to the shorter time of exposure to decay. Additionally, ancient DNA from the archaeological site exhibited greater degradation, as expected, due to its greater fragmentation. According to higher DNA yield, the post-World War II patellae exhibited higher STR typing success. The findings enhance our understanding of skeletal DNA preservation and offer practical implications for forensic and archaeological investigations, particularly in post-World War II mass graves and diverse burial conditions.

## Electronic supplementary material

Below is the link to the electronic supplementary material.

Supplementary Material 1

Supplementary Material 2

## Data Availability

The datasets generated during and/or analysed during the current study are available from the corresponding author upon reasonable request. The data are not publicly available due to privacy or ethical restrictions.
