# Supplementary material for: Patellae as a source of DNA in forensic and archaeological analysis
Source: Int J Legal Med. 2024 Nov 8;139(2):473–82. doi: 10.1007/s00414-024-03363-4 (PMC11850498; doi:10.1007/s00414-024-03363-4)
Supplement: Supplementary file 2 — Supplementary Material 2 [file 414_2024_3363_MOESM2_ESM.docm]

**METHODS**

According to the research problem and the literature review the following research hypotheses were formulated:

Hypothesis 1. There are statistically significant differences in the patellae from archaeological site Črnomelj and WWII mass grave in the amount of the DNA - expressed in ng DNA/g of bone.

Hypothesis 2. There are statistically significant differences in the patellae from archaeological site Črnomelj and WWII mass grave in the degradation ratio - Auto/Deg ratio of the DNA.

Hypothesis 3. There are statistically significant differences in the patellae from archaeological site Črnomelj and WWII mass grave in the successfully amplified loci - STR.

The normality and homogeneity of variance was tested using the Kolmogorov–Smirnov test (with Lilliefors significance correction). The research hypotheses were tested using the 95 % confidence intervals for means or medians, as suggested as an appropriate measure for testing the differences among groups, especially in medical studies ^1–3^, using the computer program IBM SPSS Statistics for Windows, version 28.0 (Statistical Package for the Social Sciences Inc., Chicago, IL, USA). As sample size is relatively small, confidence intervals can have limited power to detect significant differences ^1^. Thus, formulated hypotheses were also tested using p values. Significance was set as p≤0.05.

In the database there were data obtained from bones of 45 individuals, 25 from Črnomelj and 20 from World War II mass grave.

There was one case from Črnomelj and one from WWII mass grave, with no data for neither of the quantitative variables (amount of DNA expressed in ng DNA/g of bone and Auto/Deg ratio). Since there was not enough data, these samples were omitted from the database. There were four cases, three from Črnomelj and one from the WWII mass grave, in which the amount of DNA expressed in ng DNA/g of bone was obtained while the value for the Auto/Deg ratio was not possible to calculate because no Deg targets were amplified. In the first phase the Auto/Deg ratio for these four cases was calculated, separately for Črnomelj and WWII, using the following formula: value = Max + SD. Missing values of the degradation ratio were set to 149.23 (122.65+26.58) for Črnomelj and 9.42 (8.07 + 1.35) for WWII. The calculated values were saved in the database.

Kolmogorov–Smirnov test showed that data is not normally distributed. Thus, non-parametric Mann-Whitney tests were performed, and medians used for the confidence intervals.

**RESULTS**

Based on the results of Independent-sample median test and confidence intervals, all three hypotheses should be rejected. Results suggest significant differences (p<0.001) in the amount of DNA extracted, significant differences (p<0.001) in the degradation ratio and significant differences in STR (p<0.001) among patellae from Črnomelj and WWII mass grave. Amount of DNA extracted was higher in patellae from WWII mass grave (Me=13,18, SE=2,85) than from patellae from Črnomelj (Me=2.82, SE=1.20). Degradation ratio was higher in patellae from Črnomelj (Me=40.92, SE=9.99) than from patellae from WWII mass grave (Me=5.00, SE=0.40). Number of successfully amplified loci (out of 17) was higher in patellae from WWII mass grave (Me=16.11, SE=0.9) when compared to patellae from Črnomelj (Me=7.8, SE=1.5).


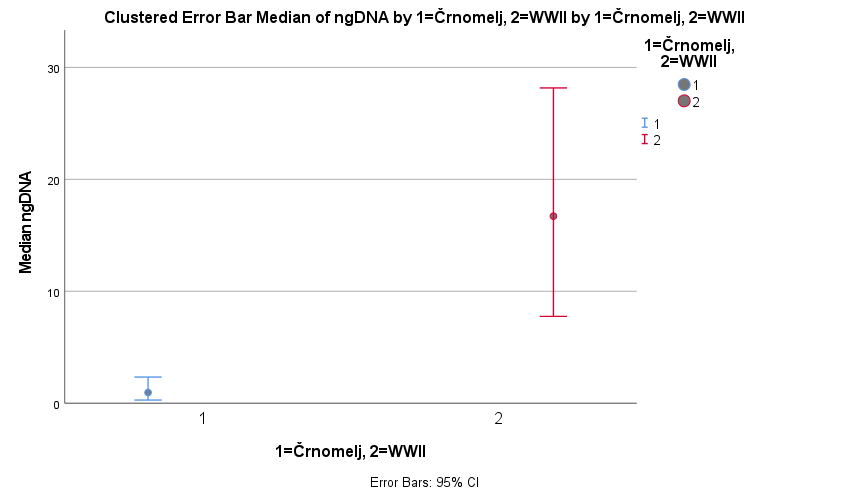


Figure 1. 95 % confidence intervals for medians for the amount of the DNA extracted from patellae (for Črnomelj and WWII mass grave, separately).


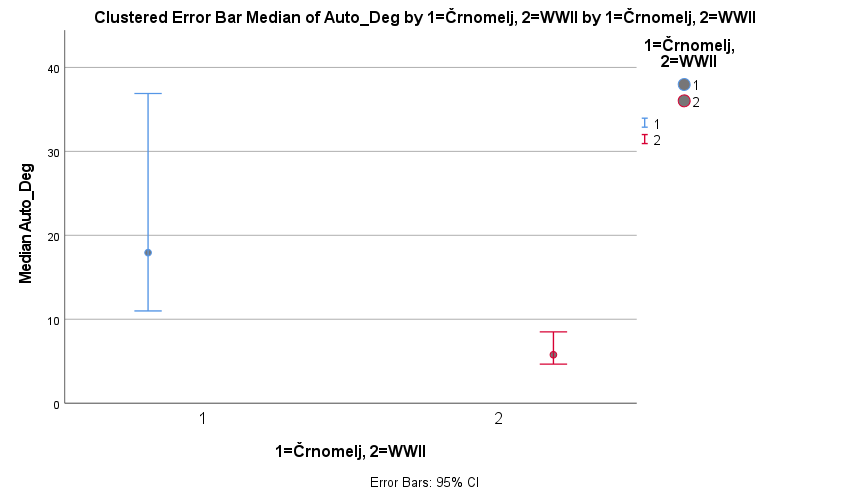


Figure 2. 95 % confidence intervals for medians for the degradation ratio of patellae (for Črnomelj and WWII mass grave, separately).


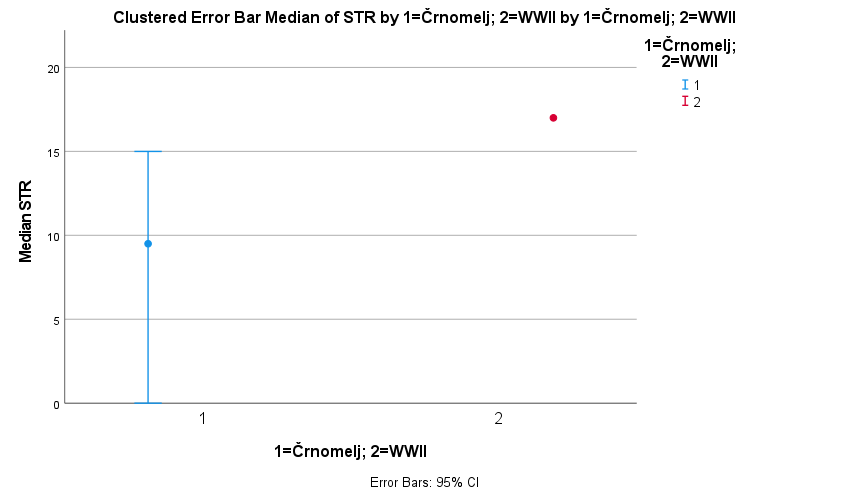


Figure 3. 95 % confidence intervals for medians for the STR of patellae (for Črnomelj and WWII mass grave, separately).

Table 1. Hypothesis test summary.

| **Hypothesis Test Summary** | | | | |
| --- | --- | --- | --- | --- |
|  | Null Hypothesis | Test | Sig.^a,b^ | Decision |
| 1 | The distribution of ngDNA is the same across categories of 1=Črnomelj; 2=WWII. | Independent-Samples Mann-Whitney U Test | <,001 | Reject the null hypothesis. |
| 2 | The distribution of Auto_Deg is the same across categories of 1=Črnomelj; 2=WWII. | Independent-Samples Mann-Whitney U Test | <,001 | Reject the null hypothesis. |
| 3 | The distribution of STR is the same across categories of 1=Črnomelj; 2=WWII. | Independent-Samples Mann-Whitney U Test | <,001 | Reject the null hypothesis. |
| a. The significance level is ,050. | | | | |
| b. Asymptotic significance is displayed. | | | | |

Table 2. Results of tests of normality.

| **Tests of Normality** | | | | | | | |
| --- | --- | --- | --- | --- | --- | --- | --- |
|  | 1=Črnomelj; 2=WWII | Kolmogorov-Smirnov^a^ | | | Shapiro-Wilk | | |
|  |  | Statistic | df | Sig. | Statistic | df | Sig. |
| STR | 1 | ,308 | 24 | <,001 | ,747 | 24 | <,001 |
|  | 2 | ,538 | 19 | <,001 | ,244 | 19 | <,001 |
| ngDNA | 1 | ,316 | 24 | <,001 | ,478 | 24 | <,001 |
|  | 2 | ,244 | 19 | ,004 | ,866 | 19 | ,012 |
| Auto_Deg | 1 | ,283 | 24 | <,001 | ,680 | 24 | <,001 |
|  | 2 | ,222 | 19 | ,014 | ,889 | 19 | ,031 |
| a. Lilliefors Significance Correction | | | | | | | |

Table 3. Results of descriptive statistics.

| **Descriptives** | | | | | |
| --- | --- | --- | --- | --- | --- |
|  | 1=Črnomelj; 2=WWII | | | Statistic | Std. Error |
| STR | 1 | Mean | | 7,79 | 1,539 |
|  |  | 95% Confidence Interval for Mean | Lower Bound | 4,61 |  |
|  |  |  | Upper Bound | 10,98 |  |
|  |  | 5% Trimmed Mean | | 7,71 |  |
|  |  | Median | | 9,50 |  |
|  |  | Variance | | 56,868 |  |
|  |  | Std. Deviation | | 7,541 |  |
|  |  | Minimum | | 0 |  |
|  |  | Maximum | | 17 |  |
|  |  | Range | | 17 |  |
|  |  | Interquartile Range | | 16 |  |
|  |  | Skewness | | -,020 | ,472 |
|  |  | Kurtosis | | -2,021 | ,918 |
|  | 2 | Mean | | 16,11 | ,895 |
|  |  | 95% Confidence Interval for Mean | Lower Bound | 14,23 |  |
|  |  |  | Upper Bound | 17,99 |  |
|  |  | 5% Trimmed Mean | | 16,95 |  |
|  |  | Median | | 17,00 |  |
|  |  | Variance | | 15,211 |  |
|  |  | Std. Deviation | | 3,900 |  |
|  |  | Minimum | | 0 |  |
|  |  | Maximum | | 17 |  |
|  |  | Range | | 17 |  |
|  |  | Interquartile Range | | 0 |  |
|  |  | Skewness | | -4,359 | ,524 |
|  |  | Kurtosis | | 19,000 | 1,014 |
| ngDNA | 1 | Mean | | 2,8179 | 1,19571 |
|  |  | 95% Confidence Interval for Mean | Lower Bound | ,3444 |  |
|  |  |  | Upper Bound | 5,2914 |  |
|  |  | 5% Trimmed Mean | | 1,7393 |  |
|  |  | Median | | ,9550 |  |
|  |  | Variance | | 34,313 |  |
|  |  | Std. Deviation | | 5,85775 |  |
|  |  | Minimum | | ,02 |  |
|  |  | Maximum | | 28,54 |  |
|  |  | Range | | 28,52 |  |
|  |  | Interquartile Range | | 2,26 |  |
|  |  | Skewness | | 4,008 | ,472 |
|  |  | Kurtosis | | 17,622 | ,918 |
|  | 2 | Mean | | 13,1784 | 2,85039 |
|  |  | 95% Confidence Interval for Mean | Lower Bound | 7,1900 |  |
|  |  |  | Upper Bound | 19,1669 |  |
|  |  | 5% Trimmed Mean | | 12,2166 |  |
|  |  | Median | | 8,6900 |  |
|  |  | Variance | | 154,369 |  |
|  |  | Std. Deviation | | 12,42454 |  |
|  |  | Minimum | | ,03 |  |
|  |  | Maximum | | 43,64 |  |
|  |  | Range | | 43,61 |  |
|  |  | Interquartile Range | | 20,59 |  |
|  |  | Skewness | | 1,125 | ,524 |
|  |  | Kurtosis | | ,418 | 1,014 |
| Auto_Deg | 1 | Mean | | 40,9317 | 9,99995 |
|  |  | 95% Confidence Interval for Mean | Lower Bound | 20,2452 |  |
|  |  |  | Upper Bound | 61,6181 |  |
|  |  | 5% Trimmed Mean | | 36,9566 |  |
|  |  | Median | | 17,9400 |  |
|  |  | Variance | | 2399,976 |  |
|  |  | Std. Deviation | | 48,98955 |  |
|  |  | Minimum | | 3,72 |  |
|  |  | Maximum | | 149,31 |  |
|  |  | Range | | 145,59 |  |
|  |  | Interquartile Range | | 33,15 |  |
|  |  | Skewness | | 1,626 | ,472 |
|  |  | Kurtosis | | 1,176 | ,918 |
|  | 2 | Mean | | 4,9942 | ,39527 |
|  |  | 95% Confidence Interval for Mean | Lower Bound | 4,1638 |  |
|  |  |  | Upper Bound | 5,8246 |  |
|  |  | 5% Trimmed Mean | | 4,8813 |  |
|  |  | Median | | 4,6600 |  |
|  |  | Variance | | 2,969 |  |
|  |  | Std. Deviation | | 1,72295 |  |
|  |  | Minimum | | 2,60 |  |
|  |  | Maximum | | 9,42 |  |
|  |  | Range | | 6,82 |  |
|  |  | Interquartile Range | | 1,85 |  |
|  |  | Skewness | | 1,242 | ,524 |
|  |  | Kurtosis | | 1,342 | 1,014 |

Table 4. Results of non-parametric Mann-Whitney test for ngDNA.

| **Ranks** | | | | | | |
| --- | --- | --- | --- | --- | --- | --- |
|  | 1=Črnomelj, 2=WWII | | N | | Mean Rank | Sum of Ranks |
| ngDNA | 1 | | 24 | | 15,94 | 382,50 |
|  | 2 | | 34 | | 39,07 | 1328,50 |
|  | Total | | 58 | |  |  |
| **Test Statistics^a^** | | | |  |  |  |
|  | | ngDNA | |  |  |  |
| Mann-Whitney U | | 82,500 | |  |  |  |
| Wilcoxon W | | 382,500 | |  |  |  |
| Z | | -5,139 | |  |  |  |
| Asymp. Sig. (2-tailed) | | <,001 | |  |  |  |
| a. Grouping Variable: 1=Črnomelj, 2=WWII | | | |  |  |  |

Table 5. Results of non-parametric Mann-Whitney test for Auto/Deg ratio.

| **Ranks** | | | | | | |
| --- | --- | --- | --- | --- | --- | --- |
|  | 1=Črnomelj, 2=WWII | | N | | Mean Rank | Sum of Ranks |
| Auto/Deg | 1 | | 24 | | 41,85 | 1004,50 |
|  | 2 | | 34 | | 20,78 | 706,50 |
|  | Total | | 58 | |  |  |
| **Test Statistics^a^** | | | |  |  |  |
|  | | Auto/Deg | |  |  |  |
| Mann-Whitney U | | 111,500 | |  |  |  |
| Wilcoxon W | | 706,500 | |  |  |  |
| Z | | -4,682 | |  |  |  |
| Asymp. Sig. (2-tailed) | | <,001 | |  |  |  |
| a. Grouping Variable: 1=Črnomelj, 2=WWII | | | |  |  |  |

1. Gardner, M. J. & Altman, D. G. Confidence intervals rather than P values: estimation rather than hypothesis testing. *Br Med J (Clin Res Ed)* **292**, 746–750 (1986).

2. Carpenter, J. & Bithell, J. Bootstrap confidence intervals: when, which, what? A practical guide for medical statisticians. *Statistics in Medicine* **19**, 1141–1164 (2000).

3. DiCiccio, T. J. & Efron, B. Bootstrap confidence intervals. *Statistical Science* **11**, 189–228 (1996).
